# Effect of Exercise Programs on Physical Performance in Community-Dwelling Older Adults with and without Frailty: Systematic Review and Meta-Analysis

**DOI:** 10.3390/geriatrics9010008

**Published:** 2024-01-08

**Authors:** Cristina Flores-Bello, Elsa Correa-Muñoz, Martha A. Sánchez-Rodríguez, Víctor Manuel Mendoza-Núñez

**Affiliations:** 1 Research Unit on Gerontology, FES Zaragoza, National Autonomous University of Mexico, Mexico City 09230, Mexico; rasguosaflores@yahoo.com.mx (C.F.-B.); elcomm_unam@yahoo.com.mx (E.C.-M.); masanrod@yahoo.com.mx (M.A.S.-R.); 2 Postgraduate Master’s and Doctorate in Nursing, National Autonomous University of Mexico, Mexico City 09230, Mexico

**Keywords:** frailty, physical exercise, physical performance, community-dwelling older adults, intrinsic capacity, functional capacity

## Abstract

Background: The measurement of physical performance constitutes an indicator of the physical functional capacity of older adults with and without frailty. Aim: To present a synthesis of knowledge on the effect of exercise programs on physical performance in older adults with and without frailty in the community. Method: A systematic review was carried out in accordance with the PRISMA-2020 criteria. The search for articles was made until 4 May 2023 in PubMed, Scopus, Web of Science, Cochrane Library, SciELO and LILACS. The outcome variable was physical performance, measured through the SPPB (Short Physical Performance Battery). The mean difference (MD) was estimated to evaluate the effect. Result: We found 2483 studies, of which 12 met the eligibility criteria for the systematic review and 9 for the meta-analysis. The effect of exercise on SPPB scores was significantly higher in the exercise group compared to control in non-frail older adults with MD = 0.51 [95% CI, 0.05 to 0.96, *p* < 0.05]. Likewise, in older adults with frailty, the effect of exercise on the global SPPB score was significantly higher in the exercise group compared to the control with MD = 0.66 [95% CI, 0.09 to 1.24, *p* < 0.05]. Conclusion: Our findings suggest that exercise programs are effective in increasing and/or maintaining physical performance in older adults with and without frailty, whose effect is more evident in older adults with frailty, probably due to the greater margin of recovery of intrinsic capacity. This systematic review shows the differentiated effect of exercise training on physical performance in older adults with and without frailty. Scientific evidence reinforces the importance of implementing physical exercise programs in all older adults, including those who are frail. However, it is necessary to specify the types and doses (duration, frequency and intensity), for individualized groups, previously grouped according to the SPPB score.

## 1. Introduction

Human aging is characterized by several physical changes, among which body composition stands out. In this sense, from the fourth decade of life, there is a decrease in muscle mass and bone tissue, as well as an increase in fat mass. These changes can contribute to the development of some chronic non-communicable diseases, such as obesity, sarcopenia and osteoporosis, which are associated with a decreased quality of life, a higher degree of dependency and a higher risk of mortality, especially when they are associated with a sedentary lifestyle and inadequate diet [1].

It has been shown that physical inactivity during aging reduces the ability to carry out activities of daily living, such as bathing, dressing, eating, personal hygiene, home cleaning, shopping and using transportation, working and going on trips, among others. In this sense, a sedentary lifestyle, in addition to causing limitations in mobility, strength and balance, increases the risk of falls and a decrease in cognitive function [2].

The World Health Organization (WHO, 2015) has defined healthy aging as “the process of developing and maintaining the functional ability that enables well-being in older age”. “Functional ability is about having the capabilities that enable all people to be and do what they have reason to value”. A combination of all the physical and mental capacities that a person has (intrinsic capacity) is required to maintain functional capacity [3].

Decreased functional capacity has been associated with a higher incidence of morbidity and mortality in old age [3]. Physical exercise training should be promoted to maintain and prolong mobility, since it is a determining factor to maintain activities of daily living and autonomy [4,5]. There is evidence of the benefits of physical exercise on physical, psychological and social functional capacity in old age [6]. The WHO (2020) in the guide on Integrated Care for Older People (ICOPE), recommends as a strategy to improve and preserve mobility in old age, the implementation of regular physical exercise programs adapted to the individual capabilities and needs of each person, including older adults with and without frailty [4].

Frailty is characterized by a decrease in physiological reserve and function in multiple organs and systems related to aging, limits the ability to cope with chronic or acute stressors, and increases the risk of adverse outcomes of health, dependence and death [7].

The measurement of physical performance is a reliable indicator of physical functional capacity, and its results allow designing physical exercise programs adapted to the individual abilities and needs of older adults, as well as evaluating their impact [8]. The Short Physical Performance Battery (SPPB) is one of the most used instruments to evaluate physical performance and functional capacity by measuring the function of the lower extremities. Decreased walking speed and sedentary lifestyle are risk factors for frailty. Therefore, measuring physical performance through the SPPB allows for the prediction of frailty, and the risk of disability, dependence, institutionalization and mortality in older people [8,9].

Some systematic reviews have been published that evaluate the effect of physical exercise programs to improve functional capacity in older adults at the community level, in which it is reported that exercise program training has a positive effect on physical performance in healthy older adults and improves the function of those who are frail [10,11]. However, these studies do not compare the results regarding the effect on older adults without or with frailty. For this reason, the objective of the present systematic review is to present a synthesis of knowledge about the effect of physical exercise programs on physical performance in community-dwelling older adults with and without frailty.

## 2. Materials and Methods

The systematic review and meta-analysis was carried out following the methodological guidelines established in the Preferred Reporting Items for Systematic Reviews (PRISMA) 2020 statement (Supplementary Materials S1) [12]. The protocol was registered in INPLASY (202350053).

### 2.1. Search Strategy

The search was carried out on the following scientific document platforms: PubMed, Scopus, Web of Science, Cochrane Library, SciELO and LILACS, until 4 May 2023. From other sources, the TesiUNAM repository was consulted. Likewise, 6 articles were selected from the studies included in the systematic review carried out by Liao et al. (2023) [10].

The keywords and search strategy were the following:

- PubMed, Web Of Science, Scopus, were: (exercise programs in community-dwelling older adults OR frailty) AND (functional capacity OR healthy aging OR Short Physical Performance Battery).
- SciELO and LILACS: (self-care program OR community program OR intrinsic capacity OR “ICOPE”) AND (functional capacity OR healthy aging OR frailty OR “SPPB”)
- Cochrane: “community program AND (functional capacity OR intrinsic capacity) AND older adult”.
- TESIUNAM: “Functional capacity” AND “older adult”.

A language filter was applied, limiting the results to articles published in English, Portuguese or Spanish, with the acceptance only of randomized controlled trials and quasi-experimental studies, in humans, and the categories of geriatrics or gerontology.

### 2.2. Inclusion and Exclusion Criteria

- Inclusion: (i) randomized clinical trials, (ii) community-dwelling older adults (≥60 years), (iii) physical exercise programs, (iv) measurement of the functional capacity of older adults with the Short Physical Performance Battery (SPPB), (v) diagnostic of frailty (7).
- Exclusion: (i) interventions in hospitals and nursing homes, (ii) cross-sectional studies, (iii) qualitative studies, (iv) protocols, reviews.

### 2.3. Article Selection

The databases were reviewed by two researchers independently, who selected the studies according to the established inclusion and exclusion criteria (CF-B and VMM-N), and discrepancies were resolved by a third researcher (MA S-R). Selection was performed manually using an Excel spreadsheet. The first step consisted of eliminating duplicate articles; subsequently, a selection was made after reading the title and abstract; finally, a thorough reading of the full text of the articles that met the eligibility criteria was carried out.

### 2.4. Data Extraction

The following data were recorded for each selected study: (i) author and year, (ii) study design, (iii) population, (iv) type of intervention, (v) measurement parameters, (vi) measurement instruments and (vii) results.

### 2.5. Evaluation of Methodological Quality

A risk of bias analysis for each study was carried out using the Cochrane Collaboration tool (RoB-2 criteria) [13]. A qualitative analysis of all documents was carried out, and only those that were methodologically similar were included for meta-analysis.

### 2.6. Data Analysis and Synthesis

Review Manager (RevMan) software version 5.4 was used [14]. The effect size was calculated using the estimation of mean differences (MD) with 95% confidence intervals (95%CI), and any score of *p* < 0.05 was considered statistically significant. The meta-analysis was carried out with the random effects method. The studies were considered to have acceptable heterogeneity with an I^2^ < 50%. A sensitivity analysis was also carried out where justified.

## 3. Results

### 3.1. Literature Search

**Figure 1:**
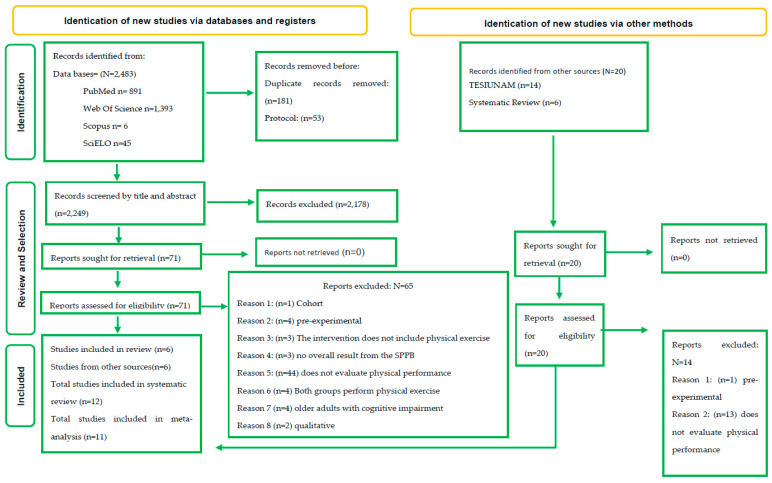
PRISMA Flowchart of the included studies.

Based on our selected keywords and search strategies, the review yielded a total of 2483 articles published until 3 May 2023. We retained 2249 after eliminating duplicates and protocols, and then excluded an additional 2178 after reviewing titles and abstracts. Among the 71 articles remaining for eligibility assessment (Figure 1), we excluded 65 for not meeting the inclusion criteria (Supplementary Materials S2), and kept a final selection of 12 trials: 6 drawn from scientific databases and 6 from the studies included by Liao et al. (2023) in their systematic review [10]. Of the 12 articles selected for review, only 9 met the meta-analysis criteria (Figure 1).

### 3.2. Assessing the Quality of the Studies

**Figure 2:**
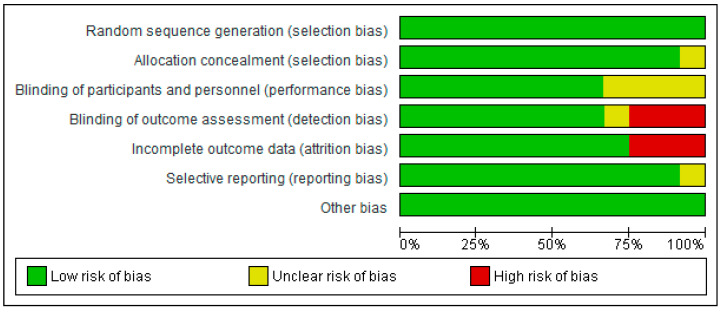
Analysis of risk of bias and methodological quality by domain.

**Figure 3:**
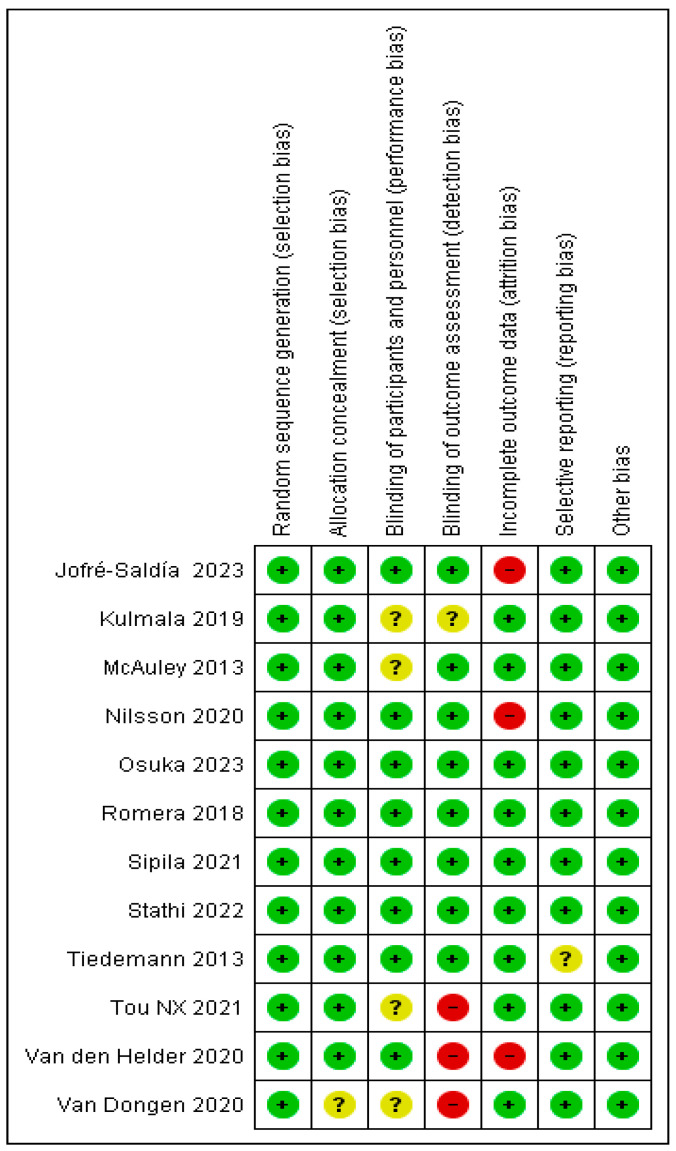
Analysis of risk of bias and methodological quality by study [15,16,17,18,19,20,21,22,23,24,25,26].

We examined the studies for possible methodological bias using Cochrane Colla-boration’s RoB-2 criteria, and found the overall methodological quality of the 12 studies to be acceptable (Figure 2 and Figure 3). Blinding of outcome assessment and incomplete outcome data were the domains most frequently showing high risk of bias, while the studies by van den Helder et al. (2020) and van Dongen et al. (2020) [15,16], showed the largest number of methodological limitations (Figure 3). Their findings refer specifically to the effect of exercise on the physical performance of older adults with frailty.

### 3.3. Study Characteristics

The 12 studies analyzed consisted of randomized clinical trials (Table 1 and Table 2) [15,16,17,18,19,20,21,22,23,24,25,26]: 2 of them combined physical exercise with nutritional and cognitive interventions [20,25]; 3 with nutritional interventions [15,16,26]; and 1 with cognitive interventions [19]. Six studies assessed the effect of physical exercise alone [17,18,21,22,23,24].

#### 3.3.1. Intervention Period

Six studies implemented interventions over a period of three months [16,21,22,23,25,26], two over six months [15,17], one over seven months [18], two over twelve months [19,24] and, finally, one over twenty-four months [20].

#### 3.3.2. Intervention Components

The types and characteristics of the exercise programs analyzed were highly heterogeneous, with activities ranging from yoga and walking to calisthenics, shoulder press, bicep curls and squats (Table 3 and Table 4). Ten studies implemented progressive, multicomponent physical training aimed at improving strength, endurance, balance and flexibility combined with two or more functions [15,16,17,18,19,20,22,23,24,26]; one implemented “Iyengar yoga”, and another, “aerobics” [21,25]. As for frequency, five studies conducted activities three times a week [17,20,22,24,26], and seven studies, twice a week on non-consecutive days [15,16,18,20,21,23,25]. The activities lasted from 40 to 60 min, except in the case of one study, in which physical exercise was performed for 10 min, one to four times daily [22].

#### 3.3.3. Intervention Venues

Seven studies conducted group activities in a gym or other community facilities equipped for their implementation [16,18,20,21,23,24,25]. In the remaining five studies, participants performed unsupervised physical activity at home [15,17,19,22,26].

#### 3.3.4. Measuring Results

All studies used the SPPB scale to assess the physical functioning of participating older adults. Five estimated the effect of physical exercise on the physical performance of those without frailty [17,18,19,20,21], while seven assessed the effect on those with frailty or pre-frailty [15,16,23,24,25,26,27]. All studies used Fried’s physical model or phenotype to determine frailty and pre-frailty.

### 3.4. The Effects of Physical Training Interventions on the Performance of Older Adults

Our meta-analysis indicated that, in contrast to the control groups, the intervention groups of older adults with and without frailty experienced a statistically significant increase in their SPPB scores.

#### 3.4.1. The Effect of Exercise on Physical Performance in Older Adults without Frailty

**Figure 4:**
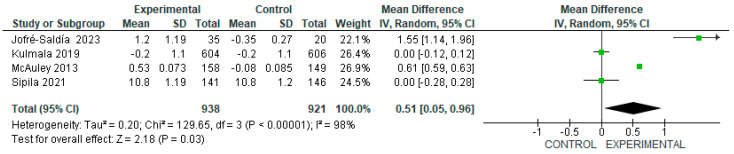
Effect of exercise programs on physical performance in older people without frailty [17,18,19,20].

Four of the eleven studies were included in the meta-analysis, to evaluate the effect of exercise on physical performance, using the SPPB in older adults without a frailty scale score. The intervention group samples were n = 938, and the control group samples were n = 921. Although, the effect of exercise proved significantly higher in the intervention as opposed to control samples, with an MD of 0.51 [95% CI, 0.05 to 0.96, *p* < 0.05]. It is important to bear in mind the wide heterogeneity among studies (I^2^ = 98%, *p* < 0.00001), in addition to the daily physical activity carried out by the subjects in the control group (Figure 4).

#### 3.4.2. The Effect of Exercise on Physical Performance in Frail Older Adults

**Figure 5:**
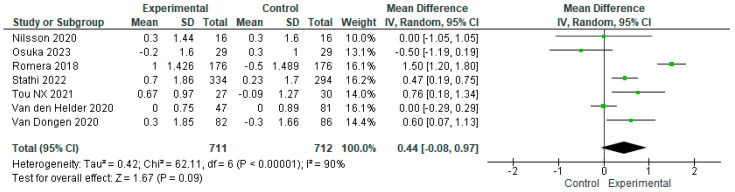
Effect of exercise programs on physical performance in frail older people [15,16,22,23,24,25,26].

Seven of the twelve studies meeting our eligibility criteria used the SPPB scale to assess the effect of exercise on the physical performance of older adults with frailty. The intervention and control group samples totaled n = 711 and n = 712, respectively. No statistically significant differences were observed in the overall effect of exercise on the physical performance of either sample [MD = 0.44.95%CI, −0.08 to 0.97, *p* = 0.09). However, as previously mentioned, the high level of heterogeneity among studies must be taken into account when considering these results (I^2^ = 90%, *p* < 0.00001) (Figure 5).

**Figure 6:**
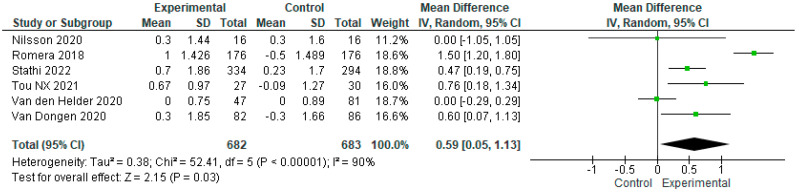
Sensitivity analysis of the effect of exercise on physical performance in frail older adults, excluding the Osuka (2023) [22] study [15,16,23,24,25,26].

**Figure 7:**
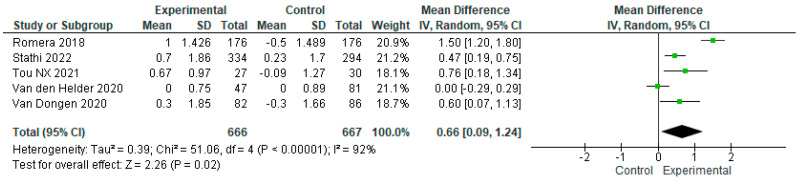
Sensitivity analysis of the effect of exercise on physical performance in frail older adults, excluding the Nilsson (2020) [26] study [15,16,23,24,25].

We conducted a sensitivity analysis excluding the study by Osuka et al. (2023), as that intervention reported a paradoxical effect in favor of the control group which the authors were unable to explain satisfactorily [22]. After this adjustment, we analyzed the data from the remaining six studies, with the intervention and control group samples totaling n = 682 and n = 683, respectively. According to the SPPB scores, the overall effect of exercise proved significantly higher in the first as compared to the second sample, with MD = 0.59 [95% CI, 0.05 to 1.13, *p* < 0.05]. Again, it is important to bear in mind the diversity of the studies (I^2^ = 90%, *p* < 0.00001) (Figure 6). Next, we conducted another sensitivity analysis excluding the Nilsson et al. (2020) trial, as that intervention was conducted exclusively with men, and the control group performed physical exercise as well [26]. After adjusting for this second exclusion, we analyzed the data from the five remaining studies, with the intervention and control group samples totaling n = 666 and n = 667, respectively. According to the results, the overall effect of exercise on the intervention group was significantly higher compared to that observed in the control group, with MD = 0.66 [95% CI, 0.09 to 1.24, *p* < 0.05]. It is important to consider the fact that the studies analyzed were highly heterogeneous (I^2^ =92%, *p* < 0.00001) (Figure 7).

#### 3.4.3. The Effect of Exercise Training Time on Physical Performance in Older Adults without Frailty

**Figure 8:**
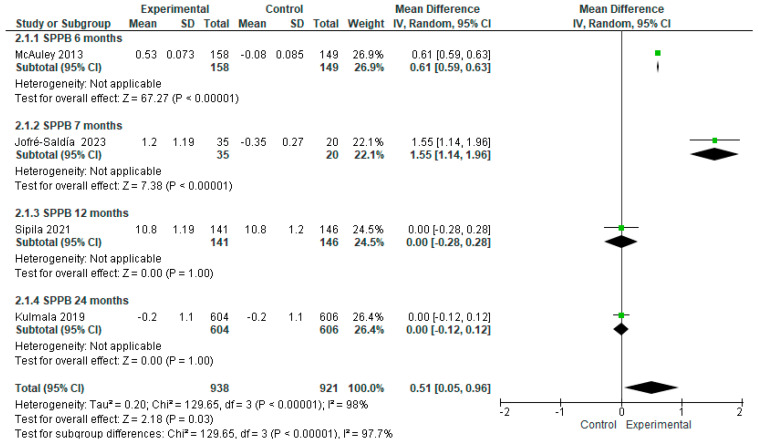
Effect of exercise training time on physical performance in older adults without frailty [17,18,19,20].

The studies of older adults without frailty featured highly variable training periods of 6, 7, 12, and 24 months [17,18,19,20]. The results profile showed a statistically significant increase in SPPB scores for periods between 6 and 7 months, but no significant effect at 12 and 24 months (Figure 8).

#### 3.4.4. The Effect of Exercise Training Time on Physical Performance in Frail Older Adults

**Figure 9:**
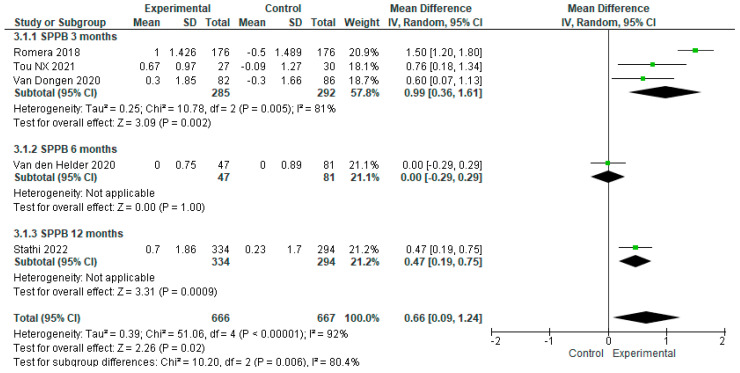
Effect of exercise training time on physical performance in frail older people [15,16,23,24,25].

Similar to those implemented for older adults without frailty, the training periods for older adults with fraility varied considerably, with timelines of 3, 6 and 12 months [15,16,23,24,25,26]. The study results profile indicated a statistically significant increase in SPPB scores at 3 months [MD = 081, 95%CI, 0.19 to 1.44, *p* = 0.01), no significant effect at 6 months, and a substantial increase at 12 months [MD = 0.47, 95%CI, 0.19 to 0.75, *p* < 0.001) (Figure 9) [15,24].

## 4. Discussion

The World Health Organization (2015) defined intrinsic capacity as “all the physical and mental capacities of an individual”, considering it a fundamental component of healthy aging [3]. For this reason, it is important to measure intrinsic capacity in healthy and independent older adults, and particularly in those with frailty, since it contributes to avoiding fractures and reducing dependency [27,28,29,30]. In this regard, the effect of exercise training on physical performance and its link with daily living activities in older adults with and without frailty is an issue of great interest for healthy aging.

For those without frailty, the studies analyzed assessed strength, balance, flexibility and aerobic endurance. They implemented two physical exercise components twice a week, in sessions of 40 to 60 min, over a period of 6 to 24 months. Meanwhile, interventions for older adults with frailty or pre-frailty featured multi-component therapeutic physical exercise training focused on balance, strength and resistance. They administered power, strength and resistance exercises progressively over a period of 3 to 12 months, in by-weekly sessions of 45 to 60 min each. It has been demonstrated that performing one type of physical exercise alone does not improve physical performance; interventions must combine aerobic activities with other types of training that develop resistance, balance and strength. To bolster the physical performance of older adults with and without frailty, physical exercise programs must ensure a proportionate combination of aerobic activity, resistance and balance training, as well as postural control [31].

Physical exercise programs must be inclusive and consider the specific conditions and requirements of the environment where participants reside. A study carried out by Fien et al. (2022) revealed the challenges facing rural communities attempting to implement physical exercise programs. Salient among them were limited access to equipment/resources, transportation and services, as well as significant costs in implementing the programs [32]. These factors highlight the need to continue assessing the effectiveness of community exercise interventions in rural areas as regards physical and functional health.

In relation to the effect of exercise on the physical performance of older adults without frailty, the studies that met our eligibility criteria recorded an adequate score before the intervention (10 to 11 on the SPPB scale). As might be expected, several authors (MacAuley et al., 2013; Jofré-Saldía et al., 2023) observed only a marginal increase in the scores. Nonetheless, it is worth mentioning that the increase either was statistically significant or persisted after the intervention (Sipilä et al., 2021; Kulmala et al., 2019) [17,18,19,20]. These results demonstrated that physical exercise exerted a positive effect on the maintenance or strengthening of intrinsic capacity, assessed through physical performance. This, in turn, translated into the maintenance or improvement of functional capacity, findings consistent with those of the systematic review conducted by Liao X, et al. (2023) [10].

Exercise training interventions have also proven effective in bolstering the physical performance of older adults with frailty. Our systematic review revealed a statistically significant increase in SPPB scores for those with and without frailty; however, the increase in the scores was higher among the former (MD = 0.66 [95% CI, 0.09 to 1.24, *p* < 0.05] vs. 0.51 [95% CI, 0.05 to 0.96, *p* < 0.05]). This suggests that there is greater room for improving or strengthening intrinsic capacity in older adults with frailty compared to those without frailty; the first lead much more sedentary lives and frequently receive anticipatory physical assistance from family members and caregivers, accentuating dependence and disability. These results are consistent with those reported in the systematic review by Salas et al. (2023). That study found greater improvement in physical performance tests on the part of older adults with frailty or pre-frailty after a physical exercise and nutrition intervention than was recorded for those without frailty. This suggests that the impact of these interventions is greater for frail as opposed to healthy older adults [27]. Likewise, in their systematic review, Haider et al. (2019) found that physical activity interventions had a positive effect on the physical performance of community-dwelling older adults with frailty or pre-frailty, increasing their muscle strength and reducing their frailty [33]. In line with these findings, international recommendations for exercise in older adults (2021) have established that exercise routines should be implemented taking into account the health characteristics of each individual; in the case of older adults with frailty, such programs should be personalized, adjusted and controlled, just as with any other medical treatment [1].

Promoting and maintaining mobility in older adults prevents care dependence; there is an inverse dose–response relationship between the time and frequency of aerobic training and the risk of physical functional limitations. In this regard, the WHO (2020) has pointed out that “doing some physical activity is better than doing nothing.” Hence, older adults should be as physically active as their functional capacity allows and limit the time they spend sitting or lying down. While they are awake, even performing general physical activities such as sweeping, climbing stairs or moving from one room to another provides health benefits. Thus, to prevent dependence and disability in older adults, any physical activity involving body movements that increase energy expenditure is valuable. Ideally, older adults should undertake physical exercise routines according to their physical condition [5].

There is no reason why older adults with frailty should not engage in physical activity and/or exercise. On the contrary, programs that include strength, balance, flexibility and aerobic exercises with a resistance component, as well as training and social support, should be implemented to encourage adherence to exercise routines and to delay the effects of aging. This is especially important during the pre-frail stage, as exercise improves functional capacity and enhances independence in performing basic activities of daily living [34].

The World Health Organization (2020) recommends performing 150 to 300 min of moderate-intensity aerobic physical activity or 75 to 150 min of vigorous aerobic physical activity per week [5].

Additionally, muscle-strengthening activities of moderate or greater intensity that involve all major muscle groups two or more days a week are recommended, as well as varied or multicomponent physical activity with an emphasis on functional balance and strength training at moderate or greater intensity, three or more days a week [5]. 

Although the benefits of physical exercise in older adults with frailty are indisputable, safe general programs that are easy to apply must be created such that they can be implemented by family or caregivers, either in group programs or at home.

Similarly, physical exercise programs aimed at older people with frailty that include nutritional intervention have been shown to increase the benefits of exercise [5,35]. In a systematic review whose objective was to determine the effectiveness of home exercise and nutrition programs on muscle quality in older adults, the authors found improvement in muscle mass, function and strength, as well as improved muscle fibers after intervention [27]. In contrast, a meta-analysis conducted by Choi et al. (2021) reported that nutritional interventions with resistance training had no additional effect on body composition, muscle strength or physical function compared to the control group who received a placebo [36]. In this regard, multimodal physical exercises should be included as part of the indications for the care of older adults with and without frailty.

In the subgroup analysis by duration of interventions in older adults without frailty, our systematic review showed an increase in SPPB scores at 6 and 7 months, and maintenance of the scores at 12 and 24 months. In the case of older adults with frailty, SPPB scores increased at 3 and 12 months, but remained unchanged at 6 months. International recommendations for exercise in older adults state that a duration of three to five months is effective in increasing the functional capacity of older adults with frailty [1]. However, given that the majority of programs in the studies analyzed were short-term, interventions conducted for longer periods of time as well as follow-up are needed to accurately describe the effectiveness of physical exercise on physical performance over time.

Regarding the method of administering physical exercise training, McAuley et al. (2013) and Sipila et al. (2021) implemented a physical exercise program at home. They delivered the training program on a DVD disk and electronic tablets, and the older adults performed the physical exercises without direct supervision by instructors or researchers. Nonetheless, participants maintained high SPPB scores until the end of the intervention, in addition to achieving acceptable adherence rates and experiencing no adverse effects related to the intervention [17,19]. Thus, it has been reported that the most viable methods for maintaining or improving strength in healthy community-dwelling older subjects consist of home physical exercise training programs. These routines are cost effective, flexible and promote independence. They can be carried out at any time of the day, and are safe and effective for older adults who have difficulty attending sessions or moving to other locations [37,38,39]. However, these results remain controversial as this approach clearly entails limitations. For example, resistance and strength exercises generally require supervision as well as specific equipment and facilities, meaning that some older adults cannot perform them at home. The social dimension is also critical, since exercises performed at home do not stimulate social participation, and thus do nothing to counter loneliness and social isolation. Additional studies concening adherence and program perseverance and its effects on physical performance are clearly necessary.

The synthesis of knowledge presented in this systematic review, shows the differentiated effect of exercise training on physical performance in older adults with and without frailty. Scientific evidence reinforces the importance of implementing physical exercise programs in all older adults, including those who are frail. However, it is necessary to specify the types and doses (time) and frequency, for individualized groups, previously grouped according to the SPPB score.

## 5. Limitations

This study suffers from several limitations. Not all available platforms of the scientific literature were explored, and our study was restricted to English language articles. We recommend conducting additional studies and expanding the process by considering a wider range of platforms as well as publications in other languages. Due to the heterogeneity of measurements and missing data, it was impossible to include all studies in the meta-analysis. The risks of physical exercise training, such as falls, injuries and cardiovascular decompensations, among others, were also not considered.

## 6. Conclusions

Overall, the present systematic review suggests that physical exercise programs are effective in increasing and/or maintaining the physical performance of older adults with and without frailty, as measured by the SPPB scale. Yet, the effect in older adults with frailty is more evident; this is as a result of the greater level of impairment and the consequent greater margin for recovery of intrinsic capacity. Multicomponent physical exercise interventions appear to be the most effective. However, there is great heterogeneity among programs (e.g., type of exercise, mode of execution, time, frequency, duration, intensity and place of intervention). We therefore recommend homogenizing program characteristics in order to determine which achieve better results in enhancing the functionality of older adults. Furthermore, physical exercise should be adapted to the functional conditions of each individual, and additional studies should be carried out, with interventions conducted over longer periods of time. Adopting these suggestions would allow for a more accurate assessment of the effectiveness of physical exercise on physical performance through time.

## Figures and Tables

**Table 1:** Effect of exercise programs on physical performance in community-dwelling older adults without frailty.

Author (Year)	Objective	Study Design	Study Population	Results/Comparison
MacAuley et al. 2013 [17]	To test the effectiveness of a home exercise program provided on DVD on the physical function of older adults.	Randomized controlled trial	307 community-dwelling older adults	SPPB EG: Pre 10.38 ± 0.118 vs. post 10.91± 0.13 CG: Pre 10.46 ± 0.14 vs. post 10.38 ± 0.13 *p* = 0.005
Jofré-Saldía et al. 2023 [18]	To evaluate the effect of a progressive multicomponent training program on functional capacity in a group of older adults in the community.	Randomized controlled trial	55 community-dwelling older adults	SPPB EG: Pre 10.60 ± 1.67 vs. post 11.80± 0.47 CG: Pre 9.45 ± 2.63 vs. post 9.10 ± 2.90 *p* = 0.000
Sipilä et al. 2021 [19]	To test whether the combination of physical and cognitive exercise has greater effects on walking speed compared to physical exercise training alone.	Randomized controlled trial	287 community-dwelling older adults	SPPB EG: Pre 10.2 ± 0.1 vs. post 10.8 ± 1.19 CG: Pre 10.1 ± 0.1 vs. Post 10.8 ± 1.2 *p* = 0.52
Kulmala et al. 2019 [20]	To investigate the effect of a multidomain lifestyle intervention on the daily functioning of older people.	Randomized controlled trial	1260 community-dwelling older adults	SPPB EG: Pre 10.8 ± 1.4 vs. post 10.8 ± 1.4 CG: Pre 10.8 ± 1.4 vs. post 10.8 ± 1.4 *p* = 0.00
Tiedemann et al. 2013 [21]	To determine the effect of an Iyengar yoga program on balance and mobility in community-dwelling older people.	Randomized controlled trial	54 community-dwelling older adults	SPPB Foot balance EG: Pre 38.9 ± 2.8 vs. post 39.7 ± 0.99 CG: Pre 38.9 ± 3.1 vs. post 38.2 ± 5.2 *p* = 0.04 Sit and stand EG: Pre 10.1 ± 3.8 vs. post 8.8 ± 2.6 CG: Pre 11.9 ± 5.2 vs. post 13.6 ± 6.1 *p* = 0.001 Walk 4 m EG: Pre 2.6 ± 0.6 vs. post 2.4 ± 0.4 CG: Pre 2.5 ± 0.6 vs. post 2.8 ± 0.6 *p* = 0.001

Abbreviations: SPPB, Short Physical Performance Battery, EG, experimental group; CG, control group.

**Table 2:** Effect of exercise programs on physical performance in community-dwelling older adults with frailty.

Author (Year)	Objective	Study Design	Study Population	Results/Comparison
Osuka et al. 2023 [22]	To determine the effectiveness of the home-based Radio Taiso exercise program in frail older adults.	Randomized controlled trial	58 community-dwelling older adults with pre-frailty and frailty	SPPB EG: pre 6.2 ± 1.9 vs. post 6.0 ± 2.0 CG: pre 6.7 ± 2.1 vs. post 7.0 ± 1.8 *p* = 0.337
Tou et al. 2021 [23]	To examine the effectiveness of a functional power exercise (FPT) program for community-dwelling pre-frail and frail older adults.	Randomized controlled trial	57 community-dwelling older adults with pre-frailty and frailty	SPPB EG: pre 10.85 ± 1.46 vs. post 11.52 ± 0.73 CG: pre 10.90 ± 1.65 vs. post 10.81 ± 2.00 *p* = 0.043
Stathi et al. 2022 [24]	To establish whether a community-based active aging intervention can prevent declines in lower extremity physical functioning in older adults at risk of mobility limitation.	Randomized controlled trial	628 community-dwelling older adults with pre-frailty and frailty	SPPB EG: pre 7.38 ± 1.58 vs. post 8.08 ± 2.87 CG: pre 7.36 ± 1.54 vs. post 7.59 ± 2.61 *p* = 0.014
Romera-Liébana et al. 2018 [25]	To evaluate the effectiveness of a multidisciplinary intervention to modify physical and cognitive frailty parameters in older people.	Randomized controlled trial	352 community-dwelling older adults with pre-frailty and frailty	SPPB EG: pre 7.1 ± 2.3 vs. post 8.1 ± 2.2 CG: pre 7.3 ± 2.4 vs. post 6.8 ± 2.3 *p* = 0.001
Nilsson et al. 2020 [26]	To examine the effects of HBRE/MIS on muscle mass, strength, and function in community-dwelling older men	Randomized controlled trial	45 varones community-dwelling older adults with pre-frailty and frailty	SPPB EG: Pre 10.3 ± 0.3 vs. post 10.6 ± 0.4 CG: Pre 11.0 ± 0.4 vs. post 11.3 ± 0.4 *p* = 0.00
Van den Helder et al. 2020 [15]	To determine the effectiveness of combined exercise (e-health + coaching) at home and a dietary protein intervention on physical performance in community-dwelling older adults.	Randomized controlled trial	128 community-dwelling older adults with pre-frailty and frailty	SPPB EG: Pre 11.19 ± 1.2 vs. post 11.2 ± 0.1 CG: Pre 11.26 ± 1.3 vs. post 11.3 ± 0.1 *p* = 0.09
Van Dongen et al. 2020 [16]	To evaluate the effectiveness of a dietary protein intervention combined with resistance exercise on physical functioning in older adults.	Randomized controlled trial	168 community-dwelling older adults with pre-frailty and frailty	SPPB EG: Pre 10.1 vs. post 10.4 CG: Pre 10.2 vs. post 9.9 *p* = 0.04

Abbreviations: SPPB, Short Physical Performance Battery, EG, experimental group; CG, control group.

**Table 3:** Characteristics of exercise in older adults without frailty.

Author (Year)	Characteristics	Doses
MacAuley et al. 2013 [17]	Multicomponent training program: Progressive strengthening exercises delivered on DVD disc to do at home, focused on flexibility, strength and balance (*FlexToBa*). Balance (standing on one foot while holding a chair, triceps extension while balancing on one leg) Flexibility (hamstring stretch). Strengthening (bicep curls and shoulder press), resistance bands are used	Time: 24 weeks. Frequency: 3 days a week. Duration: Does not mention.
Jofré-Saldía et al. 2023 [18]	Multicomponent training program: Balance and flexibility: through exercises with bosu, mini bosu, minitramp, and fitball. Resistance (elastic bands and medicine ball) Cardiorespiratory capacity: walking training in a room with dimensions of 20 m long by 10 m wide.	Time: 27 weeks divided into 3 phases of 9 weeks each. Frequency: 2 days a week. Duration: 1st phase: Strength exercises 45 min. 2nd phase: Exercises for cardiorespiratory endurance: 50 min. 3rd phase: exercises for balance and flexibility 60 min.
Sipilä et al. 2021 [19]	Multicomponent training program: Supervised training sessions and home exercises. Training periods had variations in training specificity, volume, and intensity. Pneumatic resistance training machines were used for resistance exercises. Postural balance. Muscular strength. Endurance.	Time: 12 months Frequency: 2 days per week supervised training sessions and home exercise 2 to 3 times per week. Walking and balance: 1 day per week. Stamina and balance: 1 day per week. Duration: Walk 150 min per week. Balance: 45 min. Stamina and balance: 1 h.
Kulmala et al. 2019 [20]	Multicomponent training program: Postural balance. Strength: exercises for the eight major muscle groups (knee extension and flexion, abdominal and back muscles, rotation, upper back and arm muscles, using bench press for lower extremity muscles. Individual and group aerobics such as Nordic walking, aqua gymnastics, jogging and gymnastics.	Time: 24 months. Frequency: Progressive muscle strength training: 1–3 times per week. Aerobic exercise: 2–5 times per week Duration: 40–60 min.
Tiedemann et al. 2013 [21]	Group Iyengar yoga sessions focused on standing postures to improve flexibility and muscle strength. The balance challenge increased over time by gradually increasing the difficulty of the postures performed. “Utkatasana” Chair Pose. “Trikonasana” Triangle Pose *Modification*: A block or chair is placed under the lower hand if required or the pose can be performed with back to the wall for support where needed. “Virabhadrasana 1” Warrior 1. “Virabhadarasana 2” Warrior 2. “Virabhadrasana 3” Warrior 3 *Modification*: Pose performed next to a wall for support if needed. “Vriksasana” Tree Pose *Modification*: Pose performed next to a wall for support if needed. “Adha Chandrasana” Half Moon Pose *Modification*: A block or chair is placed under the lower hand if required or the pose can be performed with back to the wall for support where needed.	Time: 12 weeks. Frequency: 2 days per week. Duration: 1 h group session.

**Table 4:** Characteristics of exercise in frail older adults.

**Author (Year)**	**Characteristics**	**Doses**
Osuka et al. 2023 [22]	Multicomponent physical exercise: Balance, strength, endurance, flexibility and coordination, broadcast daily on public radio and television. From 8 to 13 rhythmic movements with music: Radio Taiso at home. Radio Taiso no. 1 Radio Taiso no. 2 Minna no Taiso	Time: 12 weeks. Frequency: 1–4 times a day. Duration: 10 min.
Tou et al. 2021 [23]	Progressive power and balance exercises targeting upper and lower body muscles Sit to stand/squat. Knee ups (hip flexion) Calf + toe raises Knee extension Seated heel drag/hamstring curl (knee flexion) Hip extension Hip abduction For the power training, body weight and/or resistance bands were used as resistance and participants were instructed to move as fast as they can during the concentric phase and slowly during the eccentric phase (approximately 3s) of the exercise movements.	Time: 12 weeks. Frequency: 2 sessions per week. Duration: 60 min.
Stathi et al. 2022 [24]	Multicomponent training program Balance Lower extremity muscle strength Cardiorespiratory capacity Coordination and flexibility	Time: 52 weeks. Frequency: 2 sessions per week. Duration: 60 min.
Romera-Liébana et al. 2018 [25]	Aerobic exercise program.	Time: 12 weeks. Frequency: 2 sessions per week. Duration: 60 min.
Nilsson et al. 2020 [26]	Strengthening for the lower and upper body. Home resistance bands, biceps curl, triceps extension, lateral raise, seated row, bench press, sit-ups, calf raise, chair squat, knee extension, knee flexion, knee flexion were used for training. Hip and back flexion and walk at least 5000 steps on exercise days and 10,000 steps on rest days	Time: 12 weeks. Frequency: 3 days per week. Duration: does not refer.
Van den Helder et al. 2020 [15]	Multicomponent training program Progressive functional training at home. Focused on improving the frequency and intensity of functional activities of daily living (climbing stairs, getting up from a chair, and shopping). For its application, a tablet PC is provided with the personalized training program. Domain 1. Strength (strength in torso and extremities) Domain 2. Endurance (cardiorespiratory fitness) Domain 3. Flexibility (flexibility and range of motion of the torso and extremities) Domain 4. Balance and coordination (neuromotor skills)	Time: 6 months. Frequency: 2 times a week. Duration: 45 min.
Van Dongen et al. 2020 [16]	Progressive resistance exercises Leg press, leg extension, lat pulldown, upright row and chest press using leg and chest press machines to work muscle groups	Time: 3 months. Frequency: 2 times a week. Duration: 60 min.

## Data Availability

The data presented in this study are available on request from the corresponding author.

## Supplementary Materials

The following supporting information can be downloaded at: https://www.mdpi.com/article/10.3390/geriatrics9010008/s1, Materials S1: PRISMA (Systematic revision report elements and meta-analysis protocols) Verification list, 2020; Materials S2: Reasons for exclusion of full-text studies consulted in scientific databases.
