# Supplementary material for: Effect of Exercise Programs on Physical Performance in Community-Dwelling Older Adults with and without Frailty: Systematic Review and Meta-Analysis
Source: Geriatrics (Basel). 2024 Jan 8;9(1):8. doi: 10.3390/geriatrics9010008 (PMC10801556; doi:10.3390/geriatrics9010008)
Supplement: Supplementary file 1 [file geriatrics-09-00008-s001.zip › geriatrics-2675021-supplementary.pdf]

geriatrics

Supplementary Materials S1. PRISMA (Systematic revision report elements and meta-analysis protocols) Verification list, 2020

Section and Topic	Item #	Checklist item	Location where item is reported
**TITLE**			
Title	1	Identify the report as a systematic review.	1
**ABSTRACT**			
Abstract	2	See the PRISMA 2020 for Abstracts checklist.	1
**INTRODUCTION**			
Rationale	3	Describe the rationale for the review in the context of existing knowledge.	1-2
Objectives	4	Provide an explicit statement of the objective(s) or question(s) the review addresses.	2
**METHODS**			
Eligibility criteria	5	Specify the inclusion and exclusion criteria for the review and how studies were grouped for the syntheses.	3
Information sources	6	Specify all databases, registers, websites, organisations, reference lists and other sources searched or consulted to identify studies. Specify the date when each source was last searched or consulted.	2
Search strategy	7	Present the full search strategies for all databases, registers and websites, including any filters and limits used.	3
Selection process	8	Specify the methods used to decide whether a study met the inclusion criteria of the review, including how many reviewers screened each record and each report retrieved, whether they worked independently, and if applicable, details of automation tools used in the process.	3
Data collection process	9	Specify the methods used to collect data from reports, including how many reviewers collected data from each report, whether they worked independently, any processes for obtaining or confirming data from study investigators, and if applicable, details of automation tools used in the process.	3
Data items	10a	List and define all outcomes for which data were sought. Specify whether all results that were compatible with each outcome domain in each	3,5

Section and Topic	Item #	Checklist item	Location where item is reported
		study were sought (e.g. for all measures, time points, analyses), and if not, the methods used to decide which results to collect.	
	10b	List and define all other variables for which data were sought (e.g. participant and intervention characteristics, funding sources). Describe any assumptions made about any missing or unclear information.	3, 5
Study risk of bias assessment	11	Specify the methods used to assess risk of bias in the included studies, including details of the tool(s) used, how many reviewers assessed each study and whether they worked independently, and if applicable, details of automation tools used in the process.	3,14,15
Effect measures	12	Specify for each outcome the effect measure(s) (e.g. risk ratio, mean difference) used in the synthesis or presentation of results.	5,6,7
Synthesis methods	13a	Describe the processes used to decide which studies were eligible for each synthesis (e.g. tabulating the study intervention characteristics and comparing against the planned groups for each synthesis (item #5)).	3,4
	13b	Describe any methods required to prepare the data for presentation or synthesis, such as handling of missing summary statistics, or data conversions.	5,7
	13c	Describe any methods used to tabulate or visually display results of individual studies and syntheses.	5,6,7
	13d	Describe any methods used to synthesize results and provide a rationale for the choice(s). If meta-analysis was performed, describe the model(s), method(s) to identify the presence and extent of statistical heterogeneity, and software package(s) used.	15,16,17
	13e	Describe any methods used to explore possible causes of heterogeneity among study results (e.g. subgroup analysis, meta-regression).	17,18
	13f	Describe any sensitivity analyses conducted to assess robustness of the synthesized results.	15,16,17
Reporting bias assessment	14	Describe any methods used to assess risk of bias due to missing results in a synthesis (arising from reporting biases).	13,14,15
Certainty assessment	15	Describe any methods used to assess certainty (or confidence) in the body of evidence for an outcome.	4,5
**RESULTS**			
Study selection	16a	Describe the results of the search and selection process, from the number of records identified in the search to the number of studies included in	3,4,5

Section and Topic	Item #	Checklist item	Location where item is reported
		the review, ideally using a flow diagram.	
	16b	Cite studies that might appear to meet the inclusion criteria, but which were excluded, and explain why they were excluded.	3,5
Study characteristics	17	Cite each included study and present its characteristics.	6,7
Risk of bias in studies	18	Present assessments of risk of bias for each included study.	14,15
Results of individual studies	19	For all outcomes, present, for each study: (a) summary statistics for each group (where appropriate) and (b) an effect estimate and its precision (e.g. confidence/credible interval), ideally using structured tables or plots.	6-11
Results of syntheses	20a	For each synthesis, briefly summarise the characteristics and risk of bias among contributing studies.	14,15
	20b	Present results of all statistical syntheses conducted. If meta-analysis was done, present for each the summary estimate and its precision (e.g. confidence/credible interval) and measures of statistical heterogeneity. If comparing groups, describe the direction of the effect.	15-18
	20c	Present results of all investigations of possible causes of heterogeneity among study results.	15-18
	20d	Present results of all sensitivity analyses conducted to assess the robustness of the synthesized results.	15-18
Reporting biases	21	Present assessments of risk of bias due to missing results (arising from reporting biases) for each synthesis assessed.	14-15
Certainty of evidence	22	Present assessments of certainty (or confidence) in the body of evidence for each outcome assessed.	6-7
**DISCUSSION**			
Discussion	23a	Provide a general interpretation of the results in the context of other evidence.	18-21
	23b	Discuss any limitations of the evidence included in the review.	18-21
	23c	Discuss any limitations of the review processes used.	18-21
	23d	Discuss implications of the results for practice, policy, and future research.	18-21

Section and Topic	Item #	Checklist item	Location where item is reported
**OTHER INFORMATION**			
Registration and protocol	24a	Provide registration information for the review, including register name and registration number, or state that the review was not registered.	1,2
	24b	Indicate where the review protocol can be accessed, or state that a protocol was not prepared.	2
	24c	Describe and explain any amendments to information provided at registration or in the protocol.	2
Support	25	Describe sources of financial or non-financial support for the review, and the role of the funders or sponsors in the review.	22
Competing interests	26	Declare any competing interests of review authors.	22
Availability of data, code and other materials	27	Report which of the following are publicly available and where they can be found: template data collection forms; data extracted from included studies; data used for all analyses; analytic code; any other materials used in the review.	6-12

Supplementary Materials S2. Reasons for exclusion of full-text studies consulted in scientific databases

	Reference	Reason
1	Pérez Bazán, L.M.; Enfedaque-Montes, M.B.; Cesari, M.; *et al.* A community program of integrated care for frail older adults: +AGIL BARCELONA. *J. Nutr. Health Aging* **2019**, 23, 710–716.	Cohort study
2	Pothier, K.; Gagnon, C.; Fraser, S.A.; et al. A comparison of the impact of physical exercise, cognitive training and combined intervention on spontaneous walking speed in older adults. *Aging Clin. Exp. Res.* **2018**, *30*(8), 921–925.	Pre-experimental
3	Leutwyler, H.; Hubbard, E.; Cooper, B. A Group Videogame-Based Physical Activity Program Improves Walking Speed in Older Adults Living With a Serious Mental Illness. *Innov. Aging* **2022**, 6(6), igac049. doi: 10.1093/geroni/igac049.	Pre-experimental
4	Stuck, A.E.; Minder, C.E.; Peter-Wüest, I.; Gillmann, G.; Egli, C.; Kesselring, A.; Leu, R.E.; Beck, J.C. A randomized trial of in-home visits for disability prevention in community-dwelling older people at low and high risk for nursing home admission. *Arch. Intern. Med.* **2000**, 160(7),977-986.	The intervention does not include physical exercise
5	Bernardelli, G.; Roncaglione, C.; Damanti, S.; Mari, D.; Cesari, M.; Marcucci, M. Adapted physical activity to promote active and healthy ageing: the PoliFIT pilot randomized waiting list-controlled trial. *Aging Clin. Exp. Res.* **2019**, 31(4),511-518.	Does not present final results of the SPPB
6	Li, Y.; Liebel, D.V.; Friedman, B. An investigation into which individual instrumental activities of daily living are affected by a home visiting nurse intervention. *Age Ageing* **2013**, 42(1), 27-33	The intervention does not include physical activity
7	Martínez-Hernández, B.M.; Rosas-Carrasco, O.; López-Teros, M.; *et al.* Association between physical activity and physical and functional performance in non-institutionalized Mexican older adults: a cohort study. *BMC Geriatr.* **2022**, 22(1), 388. doi:10.1186/s12877-022-03083-7	Cohorte, pre-post
8	Chiu, T.Y.; Yu, H.W. Associations of multicomponent exercise and aspects of physical performance with frailty trajectory in older adults. *BMC Geriatr.* ***2022,*** 22 ,559. doi:10.1186/s12877-022-03246-6	Does not evaluate physical performance
9	Streit, I. A.; Pinto, S. S.; Silva, A. D. S.; et al. Body weight multicomponent program improves power and functional capacity responses in older adults: A quasi-experimental study. *Experimental gerontology* **2021**, 155, 111553. doi.org/10.1016/j.exger.2021.111553	Does not evaluate physical performance
10	Olsen, P. Ø.; Tully, M. A.; Del Pozo Cruz, B.; et al. Community-based exercise enhanced by a self-management programme to promote independent living in older adults: a pragmatic randomised controlled trial. *Age and ageing* **2022,** *51*(7), afac137. doi:10.1093/ageing/afac137	Intervention group and control group perform physical exercise
11	King, A. C.; Pruitt, L. A.; Phillips, W.; et al. Comparative effects of two physical activity programs on measured and perceived physical functioning and other health-related quality of life outcomes in older adults. *The journals of gerontology. Series A, Biological sciences and medical sciences* **2020**, 55(2), M74–M83. doi:10.1093/gerona/55.2.m74	Intervention group and control group perform physical exercise

12	Martel, D.; Lauzé, M.; Agnoux, A.; et al. Comparing the effects of a home-based exercise program using a gerontechnology to a community-based group exercise program on functional capacities in older adults after a minor injury. *Experimental gerontology* **2018**,108, 41–47.	Intervention group and control group perform physical exercise
13	Ambegaonkar, J. P.; Matto, H.; Ihara, E. S.; et al. Dance, Music, and Social Conversation Program Participation Positively Affects Physical and Mental Health in Community-Dwelling Older Adults: A Randomized Controlled Trial. *J. Dance Med. Sci.* **2022**, 26(4), 255–264.	The intervention does not include physical activity
14	Casas-Herrero, Á.; Sáez de Asteasu, M. L.; Antón-Rodrigo, I.; et al. Effects of Vivifrail multicomponent intervention on functional capacity: a multicentre, randomized controlled trial. *Journal of cachexia, sarcopenia and muscle* **2022**,*13*(2), 884–893.	Does not present global SPPB score
15	Sánchez-Sánchez, J. L.; de Souto Barreto, P.; Antón-Rodrigo, I.; et al. Effects of a 12-week Vivifrail exercise program on intrinsic capacity among frail cognitively impaired community-dwelling older adults: secondary analysis of a multicentre randomised clinical trial. *Age and ageing*, **2022**, 51(12), afac303. doi:10.1093/ageing/afac303	Older adults with cognitive impairment
16	Vidarte Claros, J.A.; Quintero Cruz, M.V.; Herazo Beltrán, Y. Efectos del ejercicio físico en la condición física funcional y la estabilidad en adultos mayores. *Hacia la Promoción de la Salud* **2012**, 17 (2), 79-90.	Does not evaluate physical performance
17	Pahor, M.; Guralnik, J. M.; Ambrosius, W. T.; et al. Effect of structured physical activity on prevention of major mobility disability in older adults: the LIFE study randomized clinical trial. *JAMA* **2014**, 311(23), 2387–2396.	Pre-experimental
18	Müller-Riemenschneider, F.; Petrunoff, N.; Yao, J.; et al. Effectiveness of prescribing physical activity in parks to improve health and wellbeing - the park prescription randomized controlled trial. *Int. J. Behav. Nutr. Phys. Act.* **2020**, 17(1), 42. doi:10.1186/s12966-020-00941-8	Does not evaluate physical performance
19	Sánchez-Sánchez, J. L.; de Souto Barreto, P.; Antón-Rodrigo, I.; et al. Correction to: Effects of a 12-week Vivifrail exercise program on intrinsic capacity among frail cognitively impaired community-dwelling older adults: secondary analysis of a multicentre randomised clinical trial. *Age and ageing* **2023**, 52(4), afad050. doi:10.1093/ageing/afad050	Older adults with cognitive impairment
20	Timonen, L.; Rantanen, T.; Mäkinen, E.; et al. Effects of a group-based exercise program on functional abilities in frail older women after hospital discharge. *Aging Clin. Exp. Res.* **2006**, 18(1), 50–56.	Does not evaluate physical performance
21	Buriticá-Marín, E. D.; Daza-Arana, J. E.; Jaramillo-Losada, J.; et al. Effects of a Physical Exercise Program on the Physical Capacities of Older Adults: A Quasi-Experimental Study. *Clinical interventions in aging* **2023**, 18, 273–282.	Does not evaluate physical performance
22	Esmail, A.; Vrinceanu, T.; Lussier, M.; et al. Effects of Dance/Movement Training vs. Aerobic Exercise Training on cognition, physical fitness and quality of life in older adults: A randomized controlled trial. *J. Bodyw. Mov. Ther.* **2020**, 24(1), 212–220.	Does not evaluate physical performance

23	Law, L. L. F.; Mok, V. C. T.; Yau, M. K. S.; et al. Effects of functional task exercise on everyday problem-solving ability and functional status in older adults with mild cognitive impairment-a randomised controlled trial. *Age and ageing* **2022**, 51(1), afab210. doi: 10.1093/ageing/afab210	Does not evaluate physical performance
24	Casas-Herrero, Á.; Sáez de Asteasu, M. L.; Antón-Rodrigo, I.; et al. Effects of Vivifrail multicomponent intervention on functional capacity: a multicentre, randomized controlled trial. *Journal of cachexia, sarcopenia and muscle* **2022**, 13(2), 884–893.	Older adults with cognitive impairment
25	Hébert, R.; Robichaud, L.; Roy, P. M.; et al. Efficacy of a nurse-led multidimensional preventive programme for older people at risk of functional decline. A randomized controlled trial. *Age and ageing* **2001**, 30(2), 147–153.	Does not evaluate physical performance
26	Buckley R. C. Aging Adventure Athletes Assess Achievements and Alter Aspirations to Maintain Self-Esteem. *Frontiers in psychology* **2018**, 9, 225. doi:10.3389/fpsyg.2018.00225	Cualitative
27	de Vreede, P. L.; van Meeteren, N. L.; Samson, M. M.; et al. The effect of functional tasks exercise and resistance exercise on health-related quality of life and physical activity. A randomised controlled trial. *Gerontology* **2007**, 53(1), 12–20.	Does not evaluate physical performance
28	McNamara, B.; Rosenwax, L.; Lee, E. A.; et al. Evaluation of a healthy ageing intervention for frail older people living in the community. *Australas. J. Ageing* **2016**, *35*(1), 30–35.	Does not evaluate physical performance
29	Firestone, R.; Cheng, S.; Dalhousie, S.; et al. Exploring Pasifika wellbeing: findings from a large cluster randomised controlled trial of a mobile health intervention programme. *N. Z. Med. J.* **2020**, 133(1524), 82–101.	Does not evaluate physical performance
30	Vestergaard, S.; Kronborg, C.; Puggaard, L. Home-based video exercise intervention for community-dwelling frail older women: a randomized controlled trial. *Aging Clin. Exp. Res.* **2008**, 20(5), 479–486.	Does not evaluate physical performance
31	Henwood, T. R.; Taaffe, D. R. Improved physical performance in older adults undertaking a short-term programme of high-velocity resistance training. *Gerontology* **2005**, 51(2), 108–115.	Does not evaluate physical performance
32	Gallè, F.; Di Onofrio, V.; Romano Spica, V.; et al. Improving physical fitness and health status perception in community-dwelling older adults through a structured program for physical activity promotion in the city of Naples, Italy: A randomized controlled trial. *Geriatrics & gerontology international* **2017**, 17(10), 1421–1428.	Does not evaluate physical performance
33	Capodaglio, P.; Capodaglio, E. M.; Ferri, A.; et al. Muscle function and functional ability improves more in community-dwelling older women with a mixed-strength training programme. *Age and ageing* **2005**, 34(2), 141–147.	Does not evaluate physical performance
34	Pho, A. T.; Tanner, E. K.; Roth, J.; et al. Nursing strategies for promoting and maintaining function among community-living older adults: the CAPABLE intervention. *Geriatr. Nurs.* **2012**, 33(6), 439–445.	Does not evaluate physical performance

**35**	Coelho-Júnior, H. J.; de Oliveira Gonçalvez, I.; Sampaio, R. A. C.; et al. SPeriodized and non-periodized resistance training programs on body composition and physical function of older women. *Experimental gerontology* **2019**, 121, 10–18.	Does not evaluate physical performance
36	Gothe, N. P.; Wójcicki, T. R.; Olson, E. A.; et al. Physical activity levels and patterns in older adults: the influence of a DVD-based exercise program. *J. Behav. Med.* **2015**, 38(1), 91–97.	Does not evaluate physical performance
37	Henwood, T. R.; Riek, S.; Taaffe, D. R. Strength versus muscle power-specific resistance training in community-dwelling older adults. *J. Gerontol. A. Biol. Sci. Med. Sci.* **2008**, 63(1), 83–91.	Does not evaluate physical performance
38	Franco, J. R.; Jacobs, K.; Inzerillo, C.; et al.The effect of the Nintendo Wii Fit and exercise in improving balance and quality of life in community dwelling elders. *Technol. Health Care* **2012**, 20(2), 95–115.	Does not evaluate physical performance
39	Tsai, Athena Yijung.; Sun, Ting-Shiuan.; Chen, Ming-De.; et al. The Effectiveness of Short-Term Lifestyle Intervention Programs on Physical Function, Cognition, and Quality of Life Among Community Older Adults: A Pilot Study. Topics in Geriatric Rehabilitation **2022**, 38(2), 158-164.	Does not evaluate physical performance
40	Glenn, J. M.; Gray, M.; Binns, A. The effects of loaded and unloaded high-velocity resistance training on functional fitness among community-dwelling older adults. *Age and ageing* **2015**, 44(6), 926–931.	Experimental group and control group perform physical exercise
41	Nelson, M. E.; Layne, J. E.; Bernstein, M. J.; et al. The effects of multidimensional home-based exercise on functional performance in elderly people. *The journals of gerontology. J. Gerontol. A. Biol. Sci. Med. Sci.* **2004**, 59(2), 154–160.	Does not use SPPB scale
42	Chatters, R.; Roberts, J.; Mountain, G.; et al. The long-term (24-month) effect on health and well-being of the Lifestyle Matters community-based intervention in people aged 65 years and over: a qualitative study. *BMJ open* **2017**, 7(9), e016711. doi:10.1136/bmjopen-2017-016711	Qualitative
43	Reid, K. F.; Laussen, J.; Bhatia, K.; et al. Translating the Lifestyle Interventions and Independence for Elders Clinical Trial to Older Adults in a Real-World Community-Based Setting. *J. Gerontol. A. Biol. Sci. Med. Sci.* **2019**, 74(6), 924–928.	Does not present final SPPB score
44	Wilson, M. L.; Strayer, T. E.; Davis, R.; et al. Use of an Integrated Research-Practice Partnership to Improve Outcomes of a Community-Based Strength-Training Program for Older Adults: Reach and Effect of Lifelong Improvements through Fitness Together (LIFT). *Int. J. Environ. Res. Public Health.* **2018**, 15(2), 237. doi:10.3390/ijerph15020237	Does not evaluate physical performance
45	Matthews, L.; Mitchell, F.; Stalker, K.; et al. Process evaluation of the Walk Well study: a cluster-randomised controlled trial of a community based walking programme for adults with intellectual disabilities. *BMC public health* **2016**, 16, 527. doi:10.1186/s12889-016-3179-6	Does not evaluate physical performance
46	Teixeira do Amaral, V.; Fernandes, B.; Ngomane, A. Y.; et al. Short-term community-based exercise programs in low-income older women: Does exercise intensity and modality matters?. *Experimental gerontology* **2021**, 156, 111591. doi:10.1016/j.exger.2021.111591	Does not evaluate physical performance

47	Jhaveri, S.; Romanyk, M.; Glatt, R.; et al. SMARTfit Dual-Task Exercise Improves Cognition and Physical Function in Older Adults With Mild Cognitive Impairment: Results of a Community-Based Pilot Study. *J Aging Phys. Act.* **2023**, 31(4), 621–632.	Adultos mayores con deterioro cognitivo
48	Merom, D.; Grunseit, A.; Eramudugolla, R.; et al. Cognitive Benefits of Social Dancing and Walking in Old Age: The Dancing Mind Randomized Controlled Trial. *Front. Aging Neurosci*. **2016**, 8, 26. doi:10.3389/fnagi.2016.00026	Does not evaluate physical performance
49	Anderson-Hanley, C.; Nimon, J.P.; Westen, S.C. Cognitive health benefits of strengthening exercise for community-dwelling older adults. *J. Clin. Exp. Neuropsychol*. **2010**, 32(9), 996–1001.	Does not evaluate physical performance
50	Hau, C.; Reid, K. F.; Wong, K. F.; et al. Collaborative Evaluation of the Healthy Habits Program: An Effective Community Intervention to Improve Mobility and Cognition of Chinese Older Adults Living in the U.S. *J. Nutr. Health Aging* **2016**, 20(4), 391–397.	Does not evaluate physical performance
51	Laatar, R.; Kachouri, H.; Borji, R.; et al. Combined physical-cognitive training enhances postural performances during daily life tasks in older adults. *Experimental gerontology* **2018**, 107,91–97.	Does not evaluate physical performance
52	von Berens, Å.; Fielding, R. A.; Gustafsson, T.; et al. Effect of exercise and nutritional supplementation on health-related quality of life and mood in older adults: the VIVE2 randomized controlled trial. *BMC geriatrics* **2018**, 18(1), 286. doi:10.1186/s12877-018-0976-z	Does not evaluate physical performance
53	von Bonsdorff, M.B.; Leinonen, R.; Kujala, U.M.; et al. Effect of physical activity counseling on disability in older people: a 2-year randomized controlled trial. *J. Am. Geriatr. Soc*. **2008**, 56(12), 2188–2194.	Does not evaluate physical performance
54	Dias, J. C.; Rodrigues, I. A.; Casemiro, F. G.; et al. Effects of a Health Education program on cognition, mood and functional capacity. *Rev. Bras. Enferm.* **2017**, 70(4), 814–821.	Does not evaluate physical performance
55	Conradsson, M.; Littbrand, H.; Lindelof, N.; et al. Effects of a high-intensity functional exercise programme on depressive symptoms and psychological well-being among older people living in residential care facilities: A cluster-randomized controlled trial. *Aging Ment. Health* **2010**, 14(5), 565–576.	Does not evaluate physical performance
56	von Berens, Å.; Fielding, R.A.; Gustafsson, T.; *et al.* Effect of exercise and nutritional supplementation on health-related quality of life and mood in older adults: the VIVE2 randomized controlled trial. *BMC Geriatr.* **2018**; 18: 286	Does not evaluate physical performance
57	Saito, T.; Kai, I.; Takizawa, A. Effects of a program to prevent social isolation on loneliness, depression, and subjective well-being of older adults: a randomized trial among older migrants in Japan. *Arch. Gerontol. Geriatr.* **2012**, 55(3), 539–547.	Does not evaluate physical performance
58	Karlsson, Å.; Lindelöf, N.; Olofsson, B.; et al. Effects of Geriatric Interdisciplinary Home Rehabilitation on Independence in Activities of Daily Living in Older People With Hip Fracture: A Randomized Controlled Trial. *Arch. Phys. Med. Rehabil.* **2020**, 101(4), 571–578.	Does not evaluate physical performance
59	Williams, P.; Lord, S. R. Effects of group exercise on cognitive functioning and mood in older women. *Aust. N. Z. J. Public Health* **1997**, 21(1), 45–52.	Does not evaluate physical performance

60	Wang, L.; He, L.; Tao, Y.; et al. Evaluating a Web-Based Coaching Program Using Electronic Health Records for Patients With Chronic Obstructive Pulmonary Disease in China: Randomized Controlled Trial. *J. Med. Internet. Res.* **2017**, 19(7), e264. doi: 0.2196/jmir.6743	Does not evaluate physical performance
61	Davidson, J. W.; McNamara, B.; Rosenwax, L.; et al. Evaluating the potential of group singing to enhance the well-being of older people. *Australas. J. Ageing* **2014**, 33(2), 99–104.	Does not evaluate physical performance
62	Helbostad, J. L.; Sletvold, O.; Moe-Nilssen, R. Home training with and without additional group training in physically frail old people living at home: effect on health-related quality of life and ambulation. *Clinical rehabilitation* **2004**, 18(5), 498–508.	Does not evaluate physical performance
63	Owusu, C.; Nock, N. L.; Hergenroeder, P.; et al. IMPROVE, a community-based exercise intervention versus support group to improve functional and health outcomes among older African American and non-Hispanic White breast cancer survivors from diverse socioeconomic backgrounds: Rationale, design and methods. *Contemp. Clin. Trials* **2020**, 92, 106001. https://doi.org/10.1016/j.cct.2020.106001	Does not evaluate physical performance
64	Kaushal, N.; Langlois, F.; Desjardins-Crépeau, L.; et al. Investigating dose-response effects of multimodal exercise programs on health-related quality of life in older adults. *Clin. Interv. Aging* **2019**, 14, 209–217.	Does not evaluate physical performance
65	Friedman, E. M.; Ruini, C.; Foy, R.; et al. Lighten UP! A community-based group intervention to promote psychological well-being in older adults. *Aging Ment, Health* **2017**, 21(2), 199–205.	Does not evaluate physical performance
